# Towards a potential pan-cancer prognostic signature for gene expression based on probesets and ensemble machine learning

**DOI:** 10.1186/s13040-022-00312-y

**Published:** 2022-11-03

**Authors:** Davide Chicco, Abbas Alameer, Sara Rahmati, Giuseppe Jurman

**Affiliations:** 1 Institute of Health Policy Management and Evaluation, University of Toronto, 155 College Street, M5T 3M7 Toronto, Ontario Canada; 2 Department of Biological Sciences, Kuwait University, 13 KH Firdous Street, 13060 Kuwait City, Kuwait; 3 Krembil Research Institute, 135 Nassau Street, M5T 1M8 Toronto, Ontario Canada; 4 Fondazione Bruno Kessler, Via Sommarive 18, 38123 Povo (Trento), Italy

**Keywords:** Genetic signature, Prognostic signature, Microarray, Gene expression, Cancer, Pan-cancer, Ensemble machine learning, Random forests, Pan-cancer prognosis

## Abstract

Cancer is one of the leading causes of death worldwide and can be caused by environmental aspects (for example, exposure to asbestos), by human behavior (such as smoking), or by genetic factors. To understand which genes might be involved in patients’ survival, researchers have invented *prognostic genetic signatures*: lists of genes that can be used in scientific analyses to predict if a patient will survive or not. In this study, we joined together five different prognostic signatures, each of them related to a specific cancer type, to generate a unique pan-cancer prognostic signature, that contains 207 unique probesets related to 187 unique gene symbols, with one particular probeset present in two cancer type-specific signatures (203072_at related to the MYO1E gene). We applied our proposed pan-cancer signature with the Random Forests machine learning method to 57 microarray gene expression datasets of 12 different cancer types, and analyzed the results. We also compared the performance of our pan-cancer signature with the performances of two alternative prognostic signatures, and with the performances of each cancer type-specific signature on their corresponding cancer type-specific datasets. Our results confirmed the effectiveness of our prognostic pan-cancer signature. Moreover, we performed a pathway enrichment analysis, which indicated an association between the signature genes and a protein-protein interaction analysis, that highlighted PIK3R2 and FN1 as key genes having a fundamental relevance in our signature, suggesting an important role in pan-cancer prognosis for both of them.

**Supplementary Information:**

The online version contains supplementary material available at 10.1186/s13040-022-00312-y.

## Introduction

During gene expression, the information encoded in a gene is used for the synthesis of a protein or of another functional gene product. In biological sciences, gene expression is considered as the activity of a gene: the higher its expression, the more active the gene.

The measurement of gene expression is called *gene expression profiling*, and can be performed through several techniques and technologies, including DNA microarrays. A microarray is a grid of microscope slides with thousands of tiny spots in defined positions, with each spot containing a known DNA sequence or gene [1].

Since microarrays can be generated through multiple different techniques, each gene expression dataset is associated to a particular platform on which the gene expression was measured. Each microarray platform has its own gene expression coordinates for the positions of the genes in the genome. These coordinates are indicated by probesets, that are sets of fragments of DNA known as hybridization probes [2]. Each microarray platform therefore has its own probeset system, which is usually incompatible with the probeset system of other platforms. Only platforms of the same brand can have compatible probesets between each other, and this the is case of the Affymetrix platforms GPL96, GPL97, and GPL570, for example.

In most of the cases, a probeset corresponds to one specific gene symbol. A gene symbol, instead, can be related to multiple probesets. This aspect represents a problem in bioinformatics: given a gene symbol alone, it is impossible to know to which probeset of a specific platform it refers. On the contrary, given a probeset and a platform, it is always possible to identify the related gene symbol.

To alleviate this problem, Qiyuan Li and colleagues [3] recently released Jetset, a bioinformatics tool that associate a probeset to its most likely gene symbols for some specific platforms. Even if useful, this tool does not completely solve the probeset-gene association problem.

Even though most of scientific studies still rely on gene symbols, an article by Li Li et al. [4] showed that using different probesets related to the same gene symbol would lead to different results, and advocated for the usage of probesets instead of gene symbols in bioinformatics analyses. We agree with that approach and decided to build our whole analyses on probesets rather than gene symbols.

**Genetic signatures** Groups of particular of genes together can have an important role in the characterization of diseases; these groups of genes are usually called a *genetic signatures*. When a signature can be used to differentiate patients from healthy controls, it is called a *diagnostic signature*. When a signature can be employed to differentiate survived patients from deceased patients, instead, it is called *prognostic signature*. Here we focus on the latter kind.

Cancer affects around 20 million people and causes approximately 10 million deaths globally each year [5], and the study of potential cancer signatures has been widespread in bioinformatics research worldwide. In the past, prognostic signatures have been used for specific cancer types, such as lung cancer [6] and breast cancer [7].

Here, instead, we propose a prognostic pan-cancer signature able to identify surviving patients and death-risk patients on gene expression datasets of any possible cancer types. In fact, an analysis done on multiple cancer types is called *pan-cancer* [8].

Several researchers already proposed pan-cancer signatures and pan-cancer studies in the past. Jia and colleagues [9], for example, investigated the role of a gene signature related to the COL11A1 gene for the identification of pan-cancer associated fibroblasts. Xu et al. [10] proposed a 154-gene expression pan-cancer signature derived from a transcriptome data analysis.

In another study, de Almeida and coauthors [11] proposed a centrosome amplification-related signature for clinical outcome across different cancer types. Izzi and colleagues [12] analyzed matrisome data of the extracellular matrix (ECM) to propose 29 cancer types-specific signatures. Data from the ECM were used by Yu and colleagues [13] as well to propose a 5-gene pan-cancer signature for prognosis.

Luo et al. [14] analyzed telomerase reverse transcriptase (TERT) activation data from The Cancer Genome Atlas (TCGA) to propose a TERT$^{high}$-specific mRNA expression signature for multiple cancer types.

Yuanyuan Li and coauthors [15] analyzed RNA-Seq data of the The Cancer Genome Atlas to detect a 20-gene pan-cancer signature for survival prediction. More recently, Nagy et al. [16] analyzed the same data to detect an 8-gene pan-cancer signature.

A list of prognostic genes for a specific disease can be found not only through gene expression, but by also integrating multi-omics data. Zhou et al. [17], for example, applied deep machine learning models to data of gene expression, copy number alterations (CNAs), and messenger RNA (mRNA) and detected 12 prognostic genes for breast cancer [17].

A genetic signature can be applied to a bioinformatics dataset mainly in two ways: through statistics survival models [13, 16] or supervised machine learning models [10–12, 14, 15]. Our approach belongs to the latter group: in our analysis, in fact, we employed the Random Forests [18] ensemble machine learning method. Random Forests resulted being effective in numerous computational biology studies [19] and on gene expression data in particular [20].

**Our proposed pan-cancer prognostic signature** In this study, we propose a pan-cancer prognostic signature merged from 5 already-existing cancer type-specific prognostic signatures available in the literature (breast cancer, lung cancer, prostate cancer, colon cancer, and neuroblastoma).

Three aspects make our proposed pan-cancer signature an effective tool for prognosis on gene expession data: (i) The usage of probesets instead of gene symbols; (ii) The 207 probesets derived from 5 different signatures related to a different cancer type; (iii) The application of the signature with Random Forests.

We applied our proposed pan-cancer prognostic signature on 57 gene expression datasets publicly available on GEO, made of 12 different cancer types. Moreover, to better understand the roles and the functions of the genes of our proposed signature, we then employed a gene set enrichment tool and a protein-protein interaction analysis tool, and elaborated their results [21].

Our results confirm the predictive power of our proposed pan-cancer prognostic signature, and the functional validation task unveiled relevant information about the signature genes, that can pave the way for further studies on this topic.

**This study** We organize the result of this study this way. After this Introduction, we describe the 5 original cancer type-specific signatures that we used to generate our pan-cancer signature and the 57 datasets we employed for testing (Section 2). We then describe the machine learning method we used to predict the survival of the patients and the network and pathway analysis techniques we employed for functional validation (Section 3), and the results obtained in these two steps (Section 4). Lastly, we outline some conclusions about these study and its potential future developments (Section 5).

## Datasets

In this section, we first explain how we retrieved the gene expression cancer datasets we employed in our study (Section 2.1) and then we describe how we generated our proposed pan-cancer signature (Section 2.2).

### Gene expression data of multiple cancer types

We collected gene expression datasets of the most common cancer types [5] from Gene Expression Omnibus (GEO) through Bioconductor [22, 23] packages such as GEOquery [24] and BioMart [25]. We selected only the prognostic datasets, that are the ones which include a feature about the status of the patient: alive or deceased. We filtered in only the datasets derived from platforms compatible with our pan-cancer signature probesets, that are Affymetrix Human Genome U133 platforms HG-U133A (GPL96), HG-U133B (GPL97), or HG-U133 Plus 2 (GPL570).

For this scope, we developed a Perl script [26] that retrieved 57 different prognostic cancer datasets: 17 of breast cancer, 13 of lung cancer, 10 of colorectal cancer, 5 of lymphoma, 4 of leukemia, 2 of multiple myeloma, and 1 of adrenocortical cancer, bladder cancer, neuroblastoma, ovarian cancer, skin cancer, and stomach cancer.

We included the 11 most common cancer types, plus a rare children cancer, neuroblastoma, to verify both the universal effectiveness of our pan-cancer signature in most cancer types and in one specific rare disease. We wanted to include a dataset of prostate cancer, but we could not find any prognostic one compatible with the GPL96, GPL97, or GPL570 platforms unfortunately.

We reported all the information and the quantitative characteristics of these datasets in Table 1.

**Table 1:** List of gene expression datasets employed in our analysis, sorted by cancer type

	**dataset name**	**GEO code**	**cancer type**	**neg#**	**pos#**	**samples#**	**neg%**	**pos%**
1	dataHeaton2011	GSE33371	adrenocortical cancer	16	7	23	69.57	30.43
2	dataReister2012	GSE31684	bladder cancer	38	27	65	58.46	41.54
3	dataDedeurwaerder2011	GSE20711	breast cancer	63	25	88	71.59	28.41
4	dataDesmedt2007	GSE7390	breast cancer	141	56	197	71.57	28.43
5	dataHatzis2009	GSE25066	breast cancer	152	45	197	77.16	22.84
6	dataHuang2014	GSE48390	breast cancer	11	69	80	13.75	86.25
7	dataIvshina2006	GSE4922	breast cancer	160	89	249	64.26	35.74
8	dataJezequel2015	GSE58812	breast cancer	29	77	106	27.36	72.64
9	dataKarn2011	GSE31519	breast cancer	22	41	63	34.92	65.08
10	dataKim2020	GSE135565	breast cancer	7	76	83	8.43	91.57
11	dataLin2009	GSE19697	breast cancer	6	17	23	26.09	73.91
12	dataLoi2008	GSE9195	breast cancer	63	13	76	82.89	17.11
13	dataMetzgerFilho2016	GSE88770	breast cancer	19	97	116	16.38	83.62
14	dataMiller2013	GSE45255	breast cancer	116	18	134	86.57	13.43
15	dataSabatier2010	GSE21653	breast cancer	168	83	251	66.93	33.07
16	dataSchmidt2008	GSE11121	breast cancer	154	45	199	77.39	22.61
17	dataSinn2019	GSE124647	breast cancer	43	96	139	30.94	69.06
18	dataWang2010	GSE19615	breast cancer	14	100	114	12.28	87.72
19	dataYenamandra2015	GSE61304	breast cancer	38	20	58	65.52	34.48
20	dataBeauchamp2014	GSE38832	colorectal cancer	28	93	121	23.14	76.86
21	dataChen2020	GSE161158	colorectal cancer	145	59	204	71.08	28.92
22	dataDelRoi2017	GSE72970	colorectal cancer	32	91	123	26.02	73.98
23	dataGotoh2018	GSE92921	colorectal cancer	53	5	58	91.38	8.62
24	dataMarisa2013	GSE39582	colorectal cancer	384	194	578	66.44	33.56
25	dataShinto2020	GSE143985	colorectal cancer	75	15	90	83.33	16.67
26	dataSieber2010	GSE14333	colorectal cancer	50	176	226	22.12	77.88
27	dataSmith2009a	GSE17536	colorectal cancer	73	103	176	41.48	58.52
28	dataSmith2009b	GSE17537	colorectal cancer	20	34	54	37.04	62.96
29	dataStaub2009	GSE12945	colorectal cancer	12	49	61	19.67	80.33
30	dataHerold2011	GSE22762	leukemia	26	17	43	60.47	39.53
31	dataHerold2013	GSE37642	leukemia	307	109	416	73.80	26.20
32	dataMetzeler2018	GSE12417	leukemia	103	59	162	63.58	36.42
33	dataSpivak2014	GSE47018	leukemia	7	13	20	35.00	65.00
34	dataBild2005	GSE3141	lung cancer	57	53	110	51.82	48.18
35	dataBotling2012	GSE37745	lung cancer	144	51	195	73.85	26.15
36	dataHeiskanen2015	GSE68465	lung cancer	236	207	443	53.27	46.73
37	dataKohno2011	GSE31210	lung cancer	35	191	226	15.49	84.51
38	dataMicke2011	GSE28571	lung cancer	52	47	99	52.53	47.47
39	dataPhilipsen2010	GSE19188	lung cancer	49	32	81	60.49	39.51
40	dataPintilie2013	GSE50081	lung cancer	75	105	180	41.67	58.33
41	dataPotti2006	GSE3593	lung cancer	54	143	197	27.41	72.59
42	dataRousseaux2013	GSE30219	lung cancer	199	93	292	68.15	31.85
43	dataSon2007	GSE8894	lung cancer	68	69	137	49.64	50.36
44	dataTsao2010	GSE14814	lung cancer	60	72	132	45.45	54.55
45	dataXie2011	GSE29013	lung cancer	18	36	54	33.33	66.67
46	dataZChen2020	GSE157011	lung cancer	219	264	483	45.34	54.66
47	dataIqbal2015	GSE58445	lymphoma	76	50	126	60.32	39.68
48	dataKawaguchi2012	GSE34771	lymphoma	23	10	33	69.70	30.30
49	dataLeich2009	GSE16131	lymphoma	91	88	179	50.84	49.16
50	dataLenz2008	GSE10846	lymphoma	165	249	414	39.86	60.14
51	dataVanLoo2009	GSE7788	lymphoma	6	9	15	40.00	60.00
52	dataMulligan2007	GSE9782	multiple myeloma	103	160	263	39.16	60.84
53	dataShi2010	GSE24080	multiple myeloma	78	480	558	13.98	86.02
54	dataHiyama2009	GSE16237	neuroblastoma	11	39	50	22.00	78.00
55	dataUehara2015	GSE65986	ovarian cancer	6	48	54	11.11	88.89
56	dataBogunovic2009	GSE19234	skin cancer	20	23	43	46.51	53.49
57	dataPasini2021	GSE38749	stomach cancer	9	5	14	64.29	35.71
	average			77.70	79.68	157.39	48.29	51.71
	median			53	56	121	49.64	50.36
	minimum			6	5	14	8.43	8.62
	maximum			384	480	578	91.38	91.57

All these datasets are based on the GPL96, GPL97, or GPL570 Affymetrix platforms and were downloaded from Gene Expression Omnibus (GEO) in April and May 2021.Positive sample: survived patient diagnosed with cancer. *Negative sample* deceased patient diagnosed with cancer. *pos#* number of positive samples in the dataset. *neg#* number of negative samples in the dataset. *pos%* percentage of positive samples in the dataset. *neg%* percentage of negative samples in the dataset. These prognostic datasets refer to 12 different cancer types: 17 breast cancer datasets, 13 lung cancer datasets, 10 colorectal cancer datasets, 5 lymphoma datasets, 4 leukemia datasets, 2 multiple myeloma datasets, 1 dataset for adrenocortical cancer, bladder cancer, neuroblastoma, ovarian cancer, skin cancer, and stomach cancer

### Our pan-cancer signature

To generate our proposed pan-cancer prognostic signature, we joined five different prognostic signatures available in the scientific literature. Each of these five signatures was proposed for a specific cancer type, and its probesets are compatible with the GPL96, GPL97, and GPL570 Affymetrix platforms.

In particular, the five known prognostic signatures contribute to our pan-cancer signature this way (Fig. S1):

- The sigCangelosi2020 signature for neuroblastoma, with 9 probesets (Table S1) [27] contributes to our pan-cancer signature for 4.33%;
- The sigChen2012 signature for prostate cancer, with 7 probesets (Table S1) [28] contributes to our pan-cancer signature for 3.37%;
- The sigGyorffy2013 signature for lung cancer, with 15 probesets (Table S1) [29] contributes to our pan-cancer signature for 7.21%;
- The sigHallett2012 signature for breast cancer, with 14 probesets (Table S1) [30] contributes to our pan-cancer signature for 6.73%;
- The sigVanLaar2010 signature for colon cancer, with 163 probesets (Table S2, Table S3, Table S4, and Table S5) [31, 32] contributes to our pan-cancer signature for 78.37%.

As one can notice, the sigVanLaar2010 colon cancer signature makes a large part of our signature. We decided to include signatures of common cancer types (lung cancer, breast cancer, colon cancer, and prostate cancer) plus a signature of a rare cancer (neuroblastoma) because we wanted to create a prognostic signature that could work effectively both on common cancer types and on rare cancer types.

The first step we did was to check the probesets and genes shared by multiple source signatures and therefore present multiple times in our aggregate pan-cancer signature. We used geneExpressionFromGEO [33], and BioGPS [34] for the probeset-gene annotations.

Our proposed pan-cancer signature contains the probeset 203072_at (MYO1E gene ENSG00000157483, myosin IE) [35, 36] that is present twice in our signature because it is located both in the sigVanLaar2010 signature for colorectal cancer and in the sigHallett2012 signature for breast cancer.

Our proposed signature contains 207 unique probesets related to 187 unique gene symbols in total. Some gene symbols occur multiple times:

- 3 gene symbols appear four times (CTSB, FN1, and TM4SF1);
- 7 gene symbols appear three times (ANXA2, CD55, DUSP6, KLF6, PLAUR, RPL3, and RPL3P4);
- 17 gene symbols appear twice (APOE, BGN, C10orf99, CD59, CH507-513H43, CH507-513H44, CH507-513H46, DNAJA3, IGFBP3, IRS2, NNMT, PDK1, PGK1, PRDX5, TMBIM4, TNFRSF21, VCAN, and VEGFA9);
- All the other gene symbols appear only once.

We report our pan-cancer signature in the Supplementary information (Table S1, Table S2, Table S3, Table S4, and Table S5).

## Methods

In this section, we first describe how we applied ensemble machine learning for the prediction of the survival (Section 3.1), and then we report the methods we used for the protein-protein network and pathway analysis of our pan-cancer signature genes (Section 3.2).

### Survival prediction through machine learning

In our survival prediction, we first selected the probesets of a specific signature and the survived/deceased label on each gene expression dataset, and we then applied Random Forests [18] for binary classification. Random Forests is an ensemble machine learning method based on decision trees: at each execution, it selects random subsets of the training set (randomly picking some features and some data elements), and trains a decision tree on each of these subsets. At the end of the execution, Random Forests applies each of these decision trees, which generate a binary response. Random Forests eventually applies a majority vote to these responses: if most of these decision trees generated a true outcome, Random Forests will return a true outcome; if most of these decision trees produced a false outcome instead, Random Forests will return a false results too.

Since it is known that changes in the hyper-parameters of Random Forests do not significantly affect results when the method is applied to small datasets [37], we used the default values of the R method, with 500 trees to grow [38].

In this phase we employed traditional best practices for machine learning, by splitting the data into training set (80% of the patients, randomly selected) and test set (remaining 20%) [39, 40]. For imbalanced dataset, with one of the two classes greater than 70%, we applied the ROSE oversampling technique [41]. We measured the results on the test set with several confusion matrix rates, focusing on the Matthews correlation coefficient (MCC) [42], since it is more informative than other scores [43–47]. To avoid having results due to a particular configuration of the training set and of the test set, we repeated the execution of Random Forests 100 times, and reported the average results obtained for each statistic.

Moreover, we also applied several alternative methods to Random Forests: CatBoost [48], lightGBM [49], k-Nearest Neighbors [50], and Decision Tree [51]. Since Random Forests obtained better average MCC results than the other algorithms (Supplementary File S4), we decided to base our study on Random Forests.

### Network and pathway analysis

To better understand the biological functions associated to our pan-cancer signature, we employed g:Profiler g:GOSt [52], an online web tool for functional enrichment analysis [29, 53]. g:Profiler g:GOSt reads in a list of genes and associates functions and pathways from several bioinformatics databases, such as the Gene Ontology (GO), WikiPathways (WP), and the Human Protein Atlas (HPA). g:Profiler g:GOSt associates a *p*-value to each term annotated to the input gene list. We used its g:SCS significance algorithm with 0.005 as significance threshold, as suggested by Benjamin and colleagues [54].

Knowledge about the function and the behavior of the genes of our pan-cancer signature can come from their protein-protein interactions (PPIs), too. For this reason, we looked for the protein-protein interactions associated to our pan-cancer signature on the STRING [55] database. We decided to use only the real, physical interactions provided by STRING, with confidence threshold 0.4, and to discard the predicted interactions. This way, we can focus only on the real, existing protein-protein interactions, with a high level of confidence regarding our scientific discoveries.

For network analysis, we used experimentally detected physical protein-protein interactions (PPIs) obtained from the Integrated Interactions Database (IID, June 2021 version) [56]. For pathway enrichment analysis we used two pathway sets from pathDIP (version 4) [57], core and extended pathways (predictions based on experimentally detected physical connectivity of proteins with pathway members at an association-score 0.95 and higher).

## Results

In this section, we first report and describe the results on the survival prediction obtained by our pan-cancer signature (Section 4.1), and the results obtained through the functional validation of the genes of our pan-cancer signature (Section 4.2).

### Survival prediction on all the datasets

#### Our prognostic pan-cancer signature

 We applied our pan-cancer signature with several machine learning methods: Random Forests, CatBoost, lightGBM, k-Nearest Neighbors, and Decision Tree. Among them, Random Forests obtained the highest average Matthews correlation coefficient (MCC) on average, and therefore we highlighted this method’s results. We list the results obtained with CatBoost, lightGBM, k-Nearest Neighbors, and Decision Tree in Supplementary File S4.

**Fig. 1:**
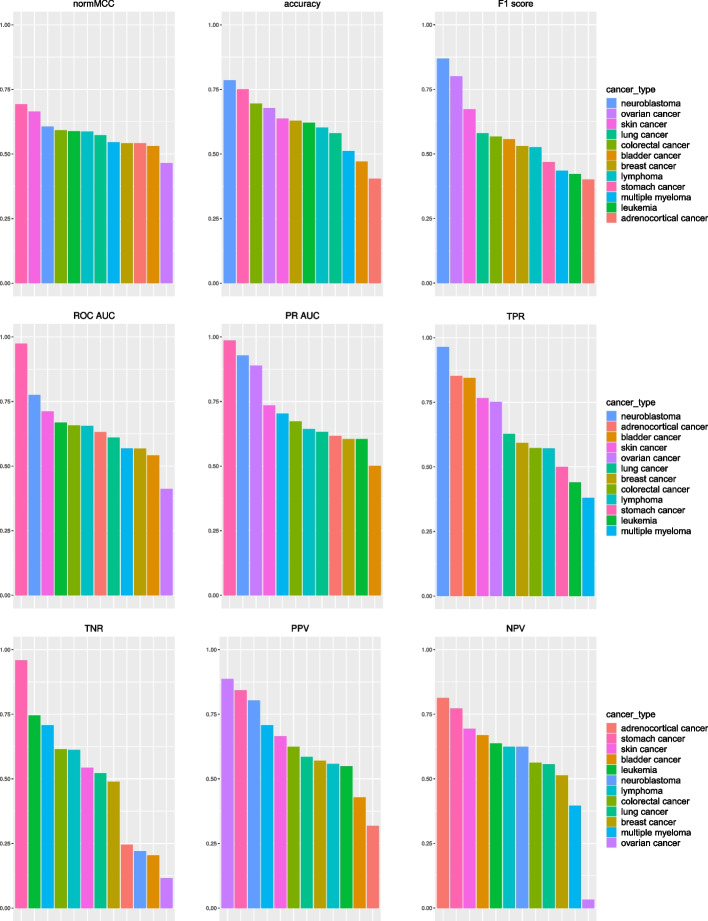
Barcharts of the average results obtained by our pan-cancer signature on each cancer type. Adrenocortical cancer: results on the dataHeaton2011 dataset. Bladder cancer: results on the dataReister2012 dataset. Breast cancer: average results on 18 breast cancer datasets. Colorectal cancer: average results on 11 colorectal cancer datasets. Leukemia: average results on 5 leukemia datasets. Lung cancer: average results on 14 lung cancer datasets. Lymphoma: average results on 6 lymphoma datasets. Multiple myeloma: average results on 3 multiple myeloma datasets. Neuroblastoma: results on the dataHiyama2009 dataset. Ovarian cancer: results on the dataUehara2015 dataset. Skin cancer: results on the dataBogunovic2009 dataset. Stomach cancer: results on the dataPasini2021 dataset. We reported the complete suvival prediction results in Table 2. normMCC: normalized Matthews correlation coefficient ($normMCC = (MCC + 1) / 2$). TPR: true positive rate, sensitivity, recall. TNR: true negative rate, specificity. PPV: positive predictive value, precision. NPV: negative predictive value. PR: precision recall curve. ROC: receiver operating characteristic curve. AUC: area under the curve. normMCC, F$_1$ score, accuracy, TPR, TNR, PPV, NPV, PR AUC, and ROC AUC have worst value 0 and best value 1. The formulas of MCC, F$_1$ score, accuracy, TPR, TNR, PPV, NPV, PR AUC and ROC AUC can be found in the Supplementary information. We report additional information about these datasets in Table 1

We report the results obtained by our prognostic signature with Random Forests on the 57 datasets in Table 2 and Fig. 1. Our pan-cancer signature achieved at least a sufficient score among the employed rates (MCC, F$_1$ score, accuracy, sensitivity, specificity, precision, negative predictive value, PR AUC, and ROC AUC) on 55 out of 57 datasets (all except the dataMicke2011 and dataLeich2009 datasets).

As expected, our signature achieved its best results among the colon cancer datasets, with 6 datasets out of 10 where the MCC is above +0.2. Our proposed signature obtained good MCC results also on the single datasets of neuroblastoma, skin cancer, and stomach cancer. It was able to generate good predictions measured with MCC on 2 leukemia datasets out of 4. Overall, regarding the Matthews correlation coefficient, our pan-cancer signature obtained sufficient results on 19 datasets out of 57, corresponding to the 33.33%.

Regarding sensitivity, our prognostic signature obtained sufficient results (TPR > 0.6) on 58.18% of the datasets, confirming its capability to recognize survived patients with cancer in the gene expression datasets. Our signature, however, obtained sufficient results for specificity only on 21.82%, showing that it is not well performing when classifying deceased patients with cancer.

We also computed the precision-recall curve AUC and the ROC curve AUC to evaluate the performances when no confusion matrix threshold is provided. Our pan-cancer signature obtained sufficient scores for the PR AUC and the ROC AUC on almost 60% of the datasets, confirming its predictive power.

Among the rankings generated with all the employed rates (Fig. 1), four cancer types result being among the first four positions on average: neuroblastoma, stomach cancer, skin cancer, and colorectal cancer. Our prognostic signature obtained more sufficient results on multiple rates on the datasets of these cancer types.

#### Other cancer type-specific signatures and pan-cancer signatures

 To further verify the predictive efficacy of our prognostic pan-cancer signature, we applied each original cancer type-specific signatures with Random Forests to each cancer type-specific dataset, and compared its results with the results obtained by our pan-cancer signature. We measured the results with the Matthews correlation coefficient.

**Table 2:** Results obtained by our pan-cancer signature on 57 gene expression datasets

	dataset name	cancer type	MCC	F_1_ score	accuracy	TPR	TNR	PPV	NPV	PR AUC	ROC AUC
1	dataHeaton2011	adrenocortical cancer	+0.082	0.401	0.404	**0.852**	0.245	0.318	**0.813**	**0.617**	**0.631**
2	dataReister2012	bladder cancer	+0.061	0.557	0.471	**0.844**	0.204	0.428	**0.669**	0.501	0.541
3	dataHatzis2009	breast cancer	**+0.252**	0.414	**0.723**	0.466	**0.801**	0.406	**0.839**	0.395	**0.697**
4	dataYenamandra2015	breast cancer	**+0.219**	0.509	0.568	**0.738**	0.490	0.417	**0.798**	0.511	**0.648**
5	dataJezequel2015	breast cancer	+0.189	**0.799**	**0.703**	**0.854**	0.308	**0.759**	0.479	**0.835**	**0.654**
6	dataSchmidt2008	breast cancer	+0.162	0.347	**0.691**	0.381	**0.784**	0.353	**0.811**	0.352	**0.643**
7	dataMiller2013	breast cancer	+0.151	0.277	**0.634**	0.561	**0.650**	0.207	**0.908**	0.267	**0.640**
8	dataSinn2019	breast cancer	+0.148	**0.898**	**0.822**	**0.923**	0.204	**0.876**	0.304	**0.903**	**0.621**
9	dataDesmedt2007	breast cancer	+0.129	0.424	0.520	**0.681**	0.462	0.317	**0.802**	0.360	0.597
10	dataKarn2011	breast cancer	+0.104	0.313	0.449	0.227	**0.871**	**0.761**	0.371	**0.740**	0.578
11	dataLoi2008	breast cancer	+0.093	0.253	**0.631**	0.433	**0.683**	0.235	**0.849**	0.311	**0.610**
12	dataIvshina2006	breast cancer	+0.070	**0.669**	0.571	**0.740**	0.321	**0.624**	0.456	**0.675**	0.563
13	dataSabatier2010	breast cancer	+0.028	0.248	**0.613**	0.210	**0.815**	0.357	**0.673**	0.366	0.527
14	dataHuang2014	breast cancer	+0.004	**0.644**	0.540	0.575	0.420	**0.789**	0.221	**0.816**	0.505
15	dataDedeurwaerder2011	breast cancer	–0.009	**0.877**	**0.788**	**0.878**	0.117	**0.881**	0.104	**0.897**	0.527
16	dataWang2010	breast cancer	–0.009	0.293	0.536	0.395	0.596	0.268	**0.725**	0.299	0.487
17	dataLin2009	breast cancer	–0.032	0.347	0.348	0.298	**0.621**	**0.695**	0.239	**0.795**	0.522
18	dataKim2020	breast cancer	–0.036	**0.891**	**0.811**	**0.879**	0.072	**0.915**	0.029	**0.898**	0.375
19	dataMetzgerFilho2016	breast cancer	–0.055	**0.832**	**0.723**	**0.839**	0.094	**0.835**	0.109	**0.851**	0.464
20	dataSieber2010	colorectal cancer	**+0.384**	**0.800**	**0.725**	**0.729**	**0.711**	**0.895**	0.442	**0.925**	**0.801**
21	dataChen2020	colorectal cancer	**+0.374**	0.482	**0.766**	0.406	**0.912**	**0.656**	**0.795**	**0.609**	**0.776**
22	dataSmith2009b	colorectal cancer	**+0.272**	**0.684**	**0.645**	**0.664**	**0.621**	**0.751**	0.513	**0.792**	**0.678**
23	dataShinto2020	colorectal cancer	**+0.240**	0.356	**0.696**	0.540	**0.739**	0.339	**0.887**	0.492	**0.723**
24	dataSmith2009a	colorectal cancer	**+0.225**	**0.666**	**0.620**	**0.672**	0.549	**0.673**	0.557	**0.708**	**0.650**
25	dataBeauchamp2014	colorectal cancer	**+0.213**	**0.801**	**0.708**	**0.798**	0.408	**0.814**	0.414	**0.842**	**0.641**
26	dataMarisa2013	colorectal cancer	+0.081	0.251	**0.645**	0.183	**0.876**	0.431	**0.682**	0.404	0.573
27	dataGotoh2018	colorectal cancer	+0.055	0.085	**0.808**	0.205	**0.863**	0.098	**0.937**	0.355	**0.789**
28	dataStaub2009	colorectal cancer	+0.002	**0.863**	**0.769**	**0.874**	0.142	**0.863**	0.116	**0.849**	0.442
29	dataDelRoi2017	colorectal cancer	–0.007	**0.677**	0.564	**0.662**	0.328	**0.721**	0.275	**0.750**	0.502
30	dataSpivak2014	leukemia	**+0.325**	0.572	**0.632**	0.525	**0.847**	**0.864**	0.524	**0.873**	**0.788**
31	dataHerold2013	leukemia	**+0.235**	0.474	**0.610**	**0.674**	0.590	0.375	**0.837**	0.460	**0.682**
32	dataMetzeler2018	leukemia	+0.136	0.309	**0.632**	0.240	**0.866**	0.518	**0.662**	0.535	**0.685**
33	dataHerold2011	leukemia	–0.001	0.187	0.537	0.189	**0.808**	0.363	**0.602**	0.479	0.526
34	dataPotti2006	lung cancer	**+0.382**	**0.752**	**0.688**	**0.652**	**0.779**	**0.898**	0.442	**0.927**	**0.780**
35	dataKohno2011	lung cancer	**+0.369**	**0.862**	**0.785**	**0.813**	**0.626**	**0.925**	0.391	**0.954**	**0.801**
36	dataRousseaux2013	lung cancer	**+0.325**	0.521	**0.711**	0.514	**0.803**	0.553	**0.783**	**0.606**	**0.679**
37	dataSon2007	lung cancer	**+0.233**	**0.615**	**0.605**	**0.666**	0.565	0.593	**0.643**	**0.634**	**0.665**
38	dataBild2005	lung cancer	+0.153	0.525	0.565	0.525	**0.627**	0.568	0.586	0.596	**0.640**
39	dataBotling2012	lung cancer	+0.129	0.418	0.522	**0.674**	0.469	0.310	**0.806**	0.415	**0.613**
40	dataHeiskanen2015	lung cancer	+0.127	**0.622**	0.535	**0.827**	0.281	0.508	**0.647**	0.584	**0.615**
41	dataPintilie2013	lung cancer	+0.070	**0.669**	0.571	**0.740**	0.321	**0.624**	0.456	**0.675**	0.563
42	dataZChen2020	lung cancer	+0.061	0.586	0.538	**0.627**	0.431	0.571	0.494	0.585	0.542
43	dataTsao2010	lung cancer	+0.055	**0.615**	0.519	**0.750**	0.289	0.541	0.539	0.572	0.531
44	dataPhilipsen2010	lung cancer	+0.045	0.375	0.547	0.386	**0.652**	0.434	**0.621**	0.491	0.545
45	dataXie2011	lung cancer	–0.016	0.548	0.496	0.508	0.480	**0.646**	0.334	**0.689**	0.491
46	dataMicke2011$^a$	lung cancer	–0.059	0.436	0.460	0.488	0.459	0.433	0.500	0.487	0.464
47	dataVanLoo2009	lymphoma	**+0.370**	**0.679**	**0.673**	**0.691**	**0.754**	**0.825**	0.584	**0.949**	**0.890**
48	dataLenz2008	lymphoma	**+0.327**	**0.755**	**0.685**	**0.805**	0.506	**0.714**	**0.630**	**0.766**	**0.723**
49	dataIqbal2015	lymphoma	+0.168	0.525	0.565	**0.646**	0.520	0.473	**0.698**	0.537	**0.623**
50	dataKawaguchi2012	lymphoma	+0.015	0.215	**0.606**	0.251	**0.762**	0.291	**0.706**	0.458	0.550
51	dataLeich2009$^a$	lymphoma	–0.014	0.458	0.481	0.466	0.520	0.486	0.500	0.503	0.494
52	dataShi2010	multiple myeloma	+0.148	**0.680**	**0.601**	**0.640**	0.514	**0.745**	0.399	**0.755**	**0.609**
53	dataMulligan2007	multiple myeloma	+0.033	0.189	0.423	0.120	**0.901**	**0.671**	0.393	**0.650**	0.529
54	dataHiyama2009	neuroblastoma	**+0.213**	**0.869**	**0.785**	**0.964**	0.220	**0.803**	**0.624**	**0.928**	**0.776**
55	dataUehara2015	ovarian cancer	–0.070	**0.800**	**0.678**	**0.752**	0.116	**0.887**	0.032	**0.889**	0.412
56	dataBogunovic2009	skin cancer	**+0.328**	**0.673**	**0.637**	**0.766**	0.543	**0.665**	**0.694**	**0.735**	**0.712**
57	dataPasini2021	stomach cancer	**+0.385**	0.469	**0.750**	0.500	**0.959**	**0.843**	**0.772**	**0.986**	**0.974**
	average	breast cancer	+0.083	0.531	**0.628**	0.593	0.489	0.570	0.513	**0.604**	0.568
	average	colorectal cancer	+0.184	0.567	**0.695**	0.573	**0.615**	**0.624**	0.562	**0.673**	**0.658**
	average	leukemia	+0.176	0.422	**0.621**	0.440	**0.745**	0.549	**0.637**	**0.604**	**0.668**
	average	lung cancer	+0.144	0.580	0.580	**0.628**	0.522	0.585	0.557	**0.632**	**0.610**
	average	lymphoma	+0.173	0.526	**0.602**	0.572	**0.612**	0.558	**0.624**	**0.643**	**0.656**
	average	multiple myeloma	+0.091	0.435	0.512	0.380	**0.708**	**0.708**	0.396	**0.703**	0.569
	% sufficient scores	all datasets	33.33%	45.45%	61.82%	58.18%	21.82%	25.45%	50.91%	58.18%	58.18%
	average	all datasets	+0.138	0.545	0.620	0.595	0.546	0.593	0.556	0.646	0.619
	median	all datasets	+0.129	0.548	0.620	0.652	0.549	0.624	0.586	0.634	0.615
	min	all datasets	–0.070	0.085	0.348	0.120	0.072	0.098	0.029	0.267	0.375
	max	all datasets	+0.385	0.898	0.822	0.964	0.959	0.925	0.937	0.986	0.974

Results obtained by the Random Forests machine learning method applied to each of the 57 prognostic cancer datasets of gene expression to predict the survival or death of the patients, sorted by cancer type and Matthews correlation coefficient. We highlighted in **bold** all the sufficient scores: $MCC \ge +0.2$, and F$_1$ score, accuracy, TPR, TNR, PPV, NPV, PR AUC, ROC AUC $\ge 0.6$. We highlighted with $^a$ the only two datasets for which all the binary classification metrics are insufficient: dataLeich2009 and dataMicke2011. MCC, F$_1$ score, accuracy, TPR, TNR, PPV, and NPV confusion matrix threshold cut-off: 0.5. *MCC* Matthews correlation coefficient. *TPR* true positive rate, sensitivity. *TNR* true negative rate, specificity. *PPV* positive predictive value, precision. *NPV* negative predictive value. *PR* precision recall curve. *ROC* receiver operating characteristic curve. *AUC* area under the curve. MCC has worst value –1 and best value +1. F$_1$ score, accuracy, TPR, TNR, PPV, NPV, PR AUC, and ROC AUC have worst value 0 and best value 1. The formulas of MCC, F$_1$ score, accuracy, TPR, TNR, PPV, NPV, PR AUC and ROC AUC can be found in the Supplementary information. % sufficient scores: percentage of datasets where the signature achieved a sufficient score (for example, our signature obtained a sufficient accuracy score on 61.82% datasets). We report additional information about these datasets in Table 1

Our pan-cancer signature outperformed the sigVanLaar2010 signature on 9 colon cancer datasets out of 10 (all except the dataSmith2009a dataset).

Our prognostic pan-cancer signature also defeated the sigHallett2021 signature on 13 breast cancer datasets out of 17 (all except the dataSinn2019, dataKarn2011, dataLin2009, and dataMetzgerFilho2018 dataset). Our proposed pan-cancer signature outplayed the sigGyorffy2013 signature on 7 lung cancer datasets out of 13 (all except the dataPhilipsen2010, dataRousseaux2013, dataSon2007, dataTsao2010, dataXie2011, dataZChen2020 dataset).

However, our prognostic pan-cancer signature was outperformed by the sigCangelosi2020 signature on the only neuroblastoma dataset. We do not have prognostic datasets of prostate cancer unfortunately so we cannot test the sigChen2012 signature singularly.

Finally, we compared the results obtained by our proposed pan-cancer signature with the results obtained by other pan-cancer signatures found in the literature: the sigNagy2021 signature [16] (Table S6) and the sigYu2021 signature [13] (Table S7).

Our pan-cancer signature outperformed the sigNagy2021 signature on 71.93% of the datasets (Supplementary File S1). Moreover, our prognostic signature defeated the sigYu2021 signature on 75.44% of the datasets (Supplementary File S2).

### Analysis of associated pathways and protein-protein interactions

#### Pathway analysis

 We input gene symbols of the probesets of our signature to pathDIP [57], and found that 139 of these genes were present in core (literature-based) pathways and were enriched in 13 pathways (Table 3). These pathways related to hypoxia-inducible factors 1 and 2 (HIF1A and HIF2A) and cell-surface signaling (ECM and integrin signalling) both of which have been shown to be implicated in cancer [58–62]. The latter also suggests potential role of protein products of these genes in interaction of cancer cells with other cells present in the tumour micro-environment. Enrichment analysis using extended pathways highlights immune system pathways (such as TLRs, interleukins, NFKB, and PDGF) as well as cell-death (apoptosis and autophagy) (Fig. S2 and Supplementary File S3).

**Table 3:** Pathways associated to our pan-cancer signature genes

			*q*-value	*q*-value
source	pathway name	*p*-value	(FDR)	(Bonferroni)
WikiPathways	Photodynamic therapy-induced	$1.05 \times 10^{-7}$	$1.82 \times 10^{-4}$	$1.82 \times 10^{-4}$
	HIF-1 survival signaling			
WikiPathways	Androgen receptor signaling	$4.43 \times 10^{-5}$	$1.54 \times 10^{-2}$	$7.67 \times 10^{-2}$
PID	Direct p53 effectors	$1.93 \times 10^{-5}$	$1.67 \times 10^{-2}$	$3.34 \times 10^{-2}$
REACTOME	Extracellular matrix organization	$3.97 \times 10^{-5}$	$1.73 \times 10^{-2}$	$6.90 \times 10^{-2}$
PID	HIF-2-alpha transcription factor network	$3.19 \times 10^{-5}$	$1.85 \times 10^{-2}$	$5.54 \times 10^{-2}$
PID	Beta1 integrin cell surface interactions	$7.02 \times 10^{-5}$	$2.03 \times 10^{-2}$	$1.22 \times 10^{-1}$
PID	Beta3 integrin cell surface interactions	$9.88 \times 10^{-5}$	$2.45 \times 10^{-2}$	$1.72 \times 10^{-1 }$
WikiPathways	Primary Focal Segmental	$1.15 \times 10^{-4}$	$2.48 \times 10^{-2}$	$1.99 \times 10^{-1}$
	Glomerulosclerosis FSGS			
PID	Alpha9 beta1 integrin signaling events	$1.31 \times 10^{-4}$	$2.52 \times 10^{-2}$	$2.27 \times 10^{-1}$
REACTOME	ECM proteoglycans	$2.01 \times 10^{-4}$	$3.18 \times 10^{-2}$	$3.50 \times 10^{-1}$
KEGG	MicroRNAs in cancer	$1.90 \times 10^{-4}$	$3.30 \times 10^{-2}$	$3.30 \times 10^{-1}$
ACSN2	MOMP_REGULATION	$3.22 \times 10^{-4}$	$4.30 \times 10^{-2}$	$5.59 \times 10^{-1}$
WikiPathways	Mammary gland development pathway	$3.12 \times 10^{-4}$	$4.51 \times 10^{-2}$	$5.42 \times 10^{-1}$
	– Puberty (Stage 2 of 4)			

List of pathways enriched with genes mapped to the probesets in the combination signature. Enrichment analysis was done using pathDIP (core pathways). *p*-value: probability value of the association. *q*-value: minimum false discovery rate at which the test may be considered significant [63]

However, despite these findings are interesting, they are highly biased due to the imbalance in the sizes of the five source signatures. In order to subdue this bias, in the next step of pathway analysis we considered genes in each of the five source signatures separately. Using PPIs available in IID [56], we identified proteins that have physical interactions to at least one protein in each source signature. Four proteins (FANCD2, EEF1A1, YWHAE, PGLS) have PPIs with at least one protein in all signatures and one protein in the breast cancer signature (ALDOC) interacts with all other four signatures. Pathway enrichment analysis of these four genes (core pathDIP) returned a list of 88 pathways. At the top of this list there is “HSF1 activation”, whose importance in several cancer types has been demonstrated [64]. The most highlighted keyword in titles of these 88 pathways are pentose phosphate, glycolysis, and fanconi all of which have strongly been linked to several cancer types [65–69].

**Fig. 2:**
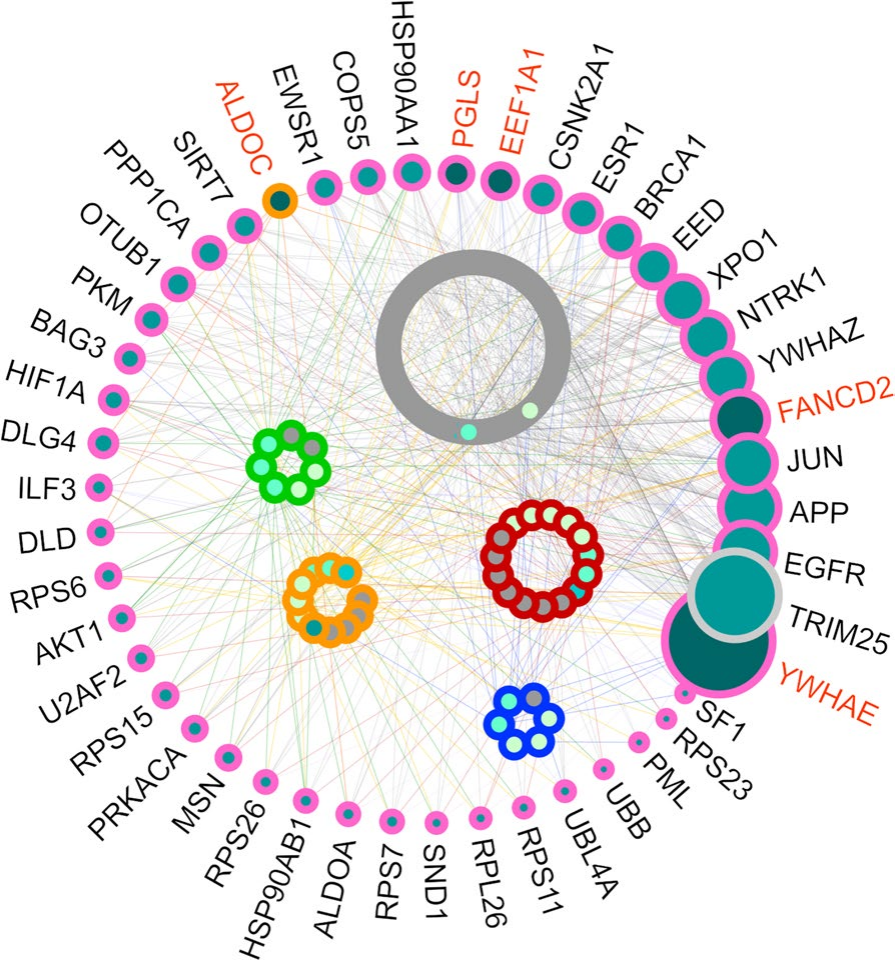
Network of integrated interactions of proteins associated to our pan-cancer signature genes. Membership of proteins that interact with protein products of genes that are members of more than three (out of five) signatures. Four proteins (FANCD2, EEF1A1, YWHAE, PGLS) have PPIs with at least one protein products of genes in all signatures and one protein in breast cancer signature (ALDOC) interacts with all other four signatures. These five genes are shown with orange labels. Genes in different signatures are shown with different outline colors: grey for colorectal cancer, red for lung cancer, carbon blue for neuroblastoma, orange for breast cancer, and green for prostate cancer. Nodes with pink outline show interacting proteins with protein products of genes of different signatures. We produced this network with IID [56]

Furthermore, we identified 42 proteins interacting with four out of five source signatures. One of these proteins (TRIM25) is a member of the colorectal cancer signature. Except for ALDOC and TRIM25, no other signature member interacts with more than three signatures. Figure 2 shows membership of proteins that interact with protein products of genes that are members of more than three (out of five) signatures.

**Fig. 3:**
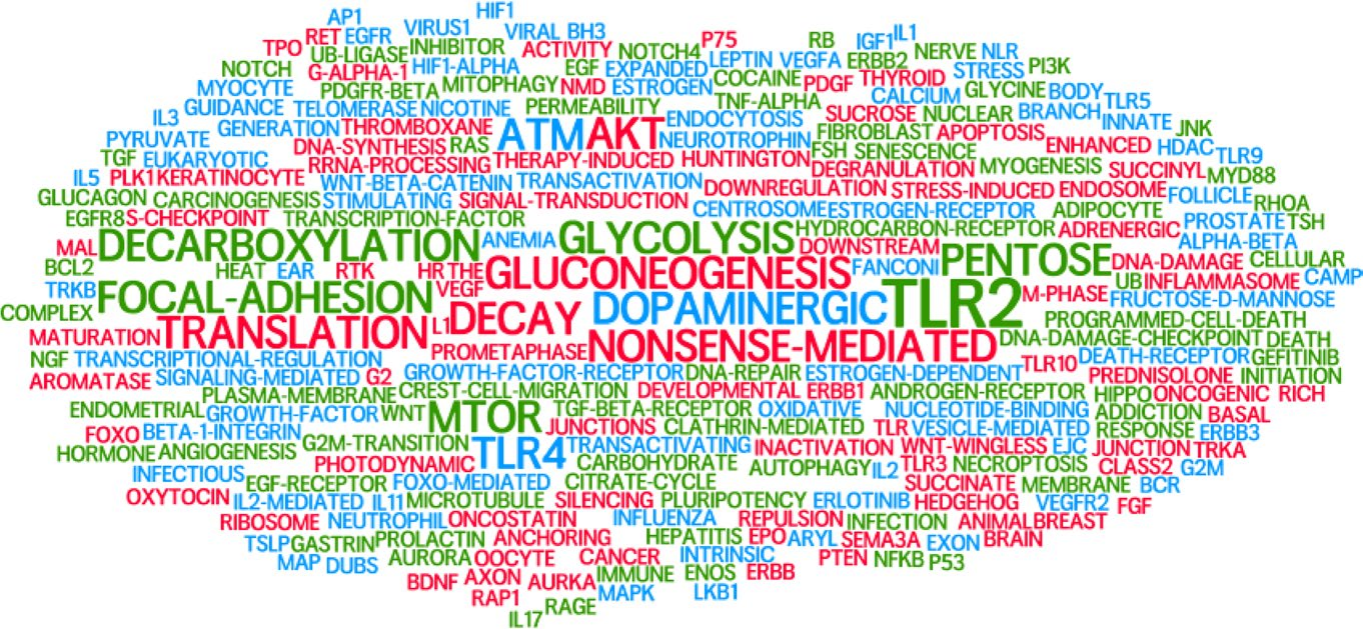
Key-term enrichment analysis. Key-term enrichment analysis of proteins that interact with protein products of genes of at least four different signatures signatures. Size of different key-terms is proportional with -log of statistical significance of appearance of each key-term in title of enriched pathways. We generated this image with pathDIP [57]

Intriguingly, the pathway enrichment analysis of these genes returned pathways that belong to main cancer hallmarks [70]. Examples of these pathways include metabolism (glycolysis, gluconeogenesis, pentose phosphate cycle, citrate-cycle), cell proliferation and maintenance (M2G, DNA-damage checkpoint, growth factors, WNT, PI3K-AKT-mTOR), cell-death (apoptosis, autophagy), immune system (TLRs, cytokine signaling, neutrophils), cell invasion (focal-adhesion, extracellular vesicle-mediated signaling, EMT), inflammation (fibroblast, integrins, TRAFs), angiogenesis (VEGF, HIF). This coverage for cancer hallmarks can partly explain reasonable performance of our combined signature on most cancer datasets (Fig. 3 and Supplementary File S3).

#### STRING protein-protein interaction networks

**Fig. 4:**
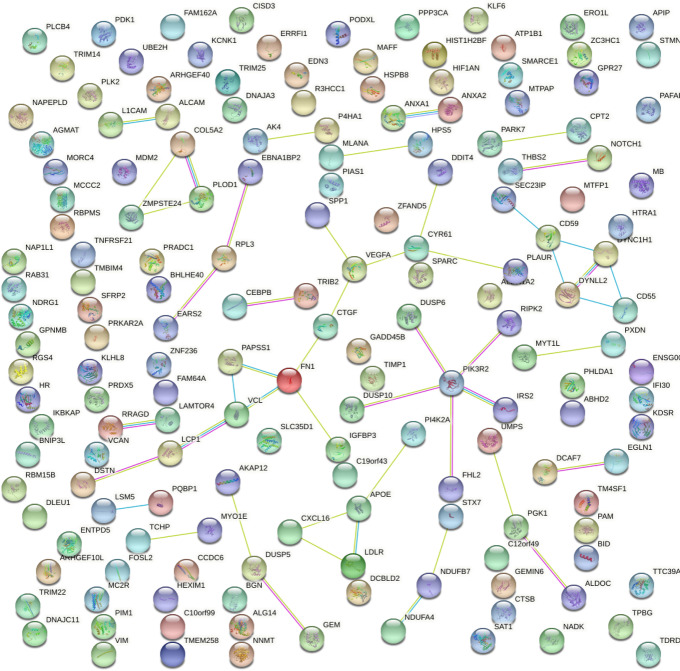
Protein-protein physical interaction network of our proposed pan-cancer signature. We generated this network with STRING [55]: each node represent a protein generated by a protein-coding gene of our proposed pan-cancer signature, and each edge represents a physical interaction between two proteins. Some nodes contain the known or predicted 3D structure of their proteins. The colors of the edges can represent several types of interactions [55]. Confidence threshold: 0.4 medium

 To better understand the relationships between the genes of our proposed pan-cancer signature, we insert it into STRING [55] and generated a network of physical protein-protein interactions (Fig. 4).

The network produced by STRING showed some interesting relationships between proteins. PIK3R2 and FN1 resulted being the proteins with the highest number of protein-protein interactions, and therefore can be considered as pan-cancer gene hubs.

The PIK3R2 gene (ENSG00000105647, phosphoinositide-3-kinase regulatory subunit 2 [71, 72]) that has 5 physical interactions in the protein-protein interaction network of STRING, which is the highest number of edges. PIK3R2 belongs to a family of genes known to be involved in pan-cancer [73]. The protein subnetwork of PIK3R2 could be used for further pan-cancer studies in the future: DUSP10, DUSP6, FHL2, IRS2, PIK3R2, and RIPK2.

The FN1 gene (ENSG00000115414, fibronectin 1 [74, 75]), that occurs 4 times in the signature (top occurrence), has 4 interactions in the STRING physical interaction network. FN1 has a key role in phosphaturic mesenchymal tumors [76]. The subnetwork of FN1 could be used for further pan-cancer studies in the future: CTGF, CYR61, DDIT4, DSTN, FN1, IGFBP3, LCP1, PAPSS1, PLAUR, SPP1, VCL, and VEGFA.

Addditionally, in the STRING physical protein-protein interaction network there are 7 proteins with 3 physical interactions, 13 proteins with 2 physical interactions, and 44 proteins with 1 physical interaction.

#### Functional enrichment analysis

**Fig. 5:**
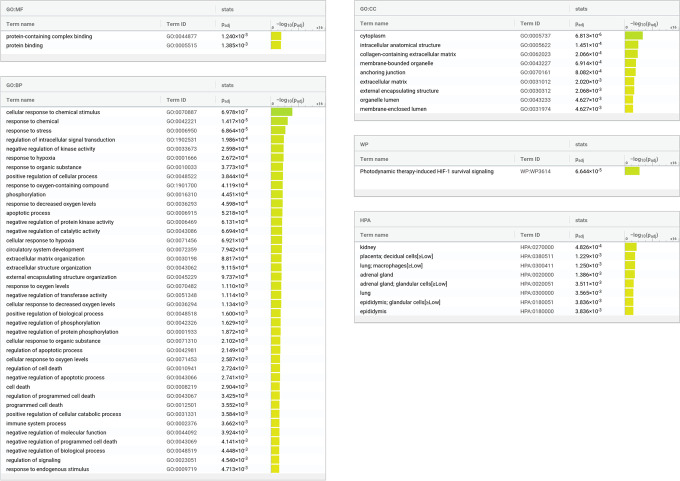
Functional annotation analysis terms associated to the genes of our proposed pancancer signature. We generated this list of functional annotations using g:Profiler g:GOSt [52] with the following options and list of abbreviations. Statistical domain scope: only annotated genes. Significance threshold: 0.005, as suggested by Benjamin and colleagues [54]. Significance method: g:SCS algorithm. GO: Gene Ontology. BP: biological process. CC: cellular component. MF: molecular function. WP: WikiPathways. TF: Transcription Factors. HPA: Human Protein Atlas

 The functional enrichment tool g:Profiler g:GOSt associated to our prognostic pan-cancer signature several pathways related to pan-cancer (Fig. 5). Gene Ontology annotations related to cancer, such as response to hypoxia apoptotic process, negative regulation of kinase activity, cellular response to hypoxia, extracellular matrix organization, extracellular structure organization, response to oxygen levels, and extracellular matrix, clearly confirm the relationship between our prognostic signature and pan-cancer. This tool also detected lung and adrenal gland as tissues from the Human Protein Atlas. g:Profiler g:GOSt associated to our pan-cancer signature several annotations related to the immune system, confirming the relevance of the genes of our pan-cancer signature in this context.

To discover additional aspects about the functional annotations related to our signature, we applied Enrichr [77] to our signature gene list. Among the annotations found by Enrichr, we found two diseases from PheWeb [78] of interest for our analysis. PheWeb associated macular degeneration to our signature gene list. We know vascular endothelial growth factor (VEGF)-A can affect cancer treatment and age-related macular degeneration [79]. PheWeb also associated lipoma of skin and subcutaneous tissue to our signature genes; a lipoma is a benign tumor made of fat. Both g:Profiler g:GOSt and Enrichr confirmed the relationship between our prognostic signature gene list and pan-cancer.

## Discussion and conclusions

In this study, we proposed a prognostic pan-cancer signature of probesets merged together from 5 different cancer type-specific signatures available in the scientific literature. Our prognostic pan-cancer signature is made of 207 unique probesets related to 187 unique gene symbols, and is based on the Affymetrix platforms GPL96, GPL97, and GPL570. We applied our proposed signature, with Random Forests and other machine learning methods, to 57 different gene expression datasets related to 12 different cancer types, and noticed that Random Forests outperformed the other algorithms with respect to the average MCC results. We analyzed the results obtained by Random Forests and our prognostic pan-cancer signature on these 57 datasets to verify its capability to classify deceased patients and survived patients. Our pan-cancer signature achieved a sufficient MCC on 33.33% of these datasets, at least one sufficient confusion matrix rate on 55 datasets out of 57, and sufficient ROC AUC and PR AUC on almost 60% of these 57 datasets.

We then compared these results with the results obtained by each specific cancer type signature on its corresponding cancer type datasets. Our signature outperformed the sigVanLaar2010 colon cancer signature on most colon cancer datasets, the sigHallett2021 breast cancer signature on most breast cancer datasets, the sigGyorffy201 lung cancer signature on most lung cancer datasets, and was outperformed by the sigCangelosi2009 neuroblastoma signature on the only neuroblastoma dataset.

Afterwards, we compared the results attained by our pan-cancer signature with the results obtained by other pan-cancer signatures that we found in the literature on the same 57 datasets: the sigNagy2021 signature and the sigYu2021 signature. Our prognostic pan-cancer signature outperformed these two signatures on more than 70% of the datasets.

These results show that, even if not perfect, the genes of our genetic signature have a relevant role in pan-cancer prognosis, and they can serve as an effective starting point for future studies on this theme. In the future, in fact, researchers can explore the genes of our pan-cancer signature to extrapolate new signatures from subgroups of the signature genes. A clear limitation of our signature is that it obtained sufficient MCC results only on 20 datasets out of 57. Our initial goal, however, was so ambitious that this outcome results being relevant in any case: we initially wanted to create a pan-cancer signature made of a list of genes able to discriminate between survived patients and deceased patients for all the possible cancer types. To this ambitious end, having a prognostic signature working well on 33.33% of the datasets represents already a sufficient and relevant result.

Additionally, as mentioned earlier, our prognostic pan-cancer signature was able to outperform other two pan-cancer signatures on most of the datasets, and almost each cancer type-specific signature on its corresponding cancer type-specific datasets. Our proposed pan-cancer signature was outplayed only by the sigCangelosi2009 neuroblastoma signature on the dataHiyama2009 neuroblastoma dataset. We believe this result is due to the orientation of our pan-cancer signature to general common cancer types, such as lung cancer, breast cancer, and colon cancer. Neuroblastoma is a rare, genetic, pediatric cancer disease, and its genetic specificity makes it different from the main cancer types such as colon cancer. We therefore believe our prognostic signature can be considered effective on common cancer types, but less effective than cancer type-specific signatures on cancer type-specific datasets of rare children cancer diseases.

Our results also confirmed the efficacy of Random Forests, a relatively-new ensemble machine learning method which has become widespread in biomedical informatics studies.

To better understand the pan-cancer role of our signature, we then investigated the pathways, the protein-protein interactions, and the functional annotations associated to our signature’s gene list.

The pathway enrichment analysis carried out with pathDIP and g:Profiler g:GOSt suggested that the genes of our signatures are related to interaction of cancer cells with each other and with other cell types present in the tumour micro-environment and to other fundamental biological aspects such as immune system and cell death. Moreover, the analysis of protein-protein interactions related to our pan-cancer signature carried out with IID highlighted the role of proteins known to be associated to several cancer types and to cancer hallmarks. The additional analysis on the protein-protein physical interactions found by STRING highlighted the proteins of the PIK3R2 (phosphoinositide-3-kinase regulatory subunit 2) and FN1 (fibronectin 1) genes as fundamental hubs in our signature, indicating an important role of these genes for pan-cancer.

Moreover, it is interesting to notice that the most relevant pathways found by pathDIP for our pan-cancer signature are known to be related to general aspect of cancer, and their association has been shown through wet lab non-computational techniques in the past: photodynamic therapy-induced HIF-1 survival signaling [80, 81], androgen receptor signaling [82], direct p53 effectors [83], HIF-2-alpha transcription factor network [84], for example.

Regarding limitations, we report that we employed here only microarray gene expression data, and did not use RNA-Seq data, which is a more modern data type. Additionally, we could not use the TCGA data [8], a dataset employed often nowadays for pan-cancer studies, because we based our study on Affymetrix probesets compatible among different GEO datasets, which would not have found direct compatibility with probesets on TCGA. For the same reason, we decided to use no data from ArrayExpress [85], which is a large alternative repository of gene expression.

In the future, we plan to use subgroups of genes indicated by the protein-protein interaction analysis as potential novel pan-cancer signatures.

## Supplementary Information

Additional file 1: Supplementary information.

Additional file 2: Supplementary File S1.

Additional file 3: Supplementary File S2.

Additional file 4: Supplementary File S3.

Additional file 5: Supplementary File S4.

## Data Availability

The datasets used in this project are publicly available on the GEO webpages listed in Table 1. Our software code is publicly available under GNU General Public License v3.0 at: https://github.com/davidechicco/pancancer_signature_on_gene_expression_data.

## Abbreviations

AUC: Area under the curve
CNAs: Copy number alterations
ECM: Extracellular matrix
FDR: False discovery rate
GEO: Gene Expression Omnibus
GO: Gene Ontology
GPL570: Affymetrix Human Genome U133 HG-U133 Plus 2
GPL96: Affymetrix Human Genome U133 HG-U133A
GPL97: Affymetrix Human Genome U133 HG-U133B
HPA: Human Protein Atlas
IID: Integrated Interactions Database
MCC: Matthews correlation coefficient
mRNA: Messenger RNA
NPV: Negative predictive value
PPI: Protein-protein interaction
PPV: Positive predictive value, precision
PR: Precision-recall
RNA: Messenger ribonucleic acid
RNA-Seq: RNA-sequencing
ROC: Receiver operating characteristic
ROSE: Random Over-Sampling Examples
STRING: Search tool for recurring instances of neighbouring genes
TCGA: The Cancer Genome Atlas
TERT: Telomerase reverse transcriptase
TNR: True negative rate, specificity
TPR: True positive rate, sensitivity, recall
WP: WikiPathways
