# Supplementary material for: Towards a potential pan-cancer prognostic signature for gene expression based on probesets and ensemble machine learning
Source: BioData Min. 2022 Nov 3;15:28. doi: 10.1186/s13040-022-00312-y (PMC9632055; doi:10.1186/s13040-022-00312-y)
Supplement: Supplementary file 1 — Additional file 1: Supplementary information. [file 13040_2022_312_MOESM1_ESM.pdf]

Supplementary information

Our proposed pan-cancer signature

Table S1: *Our proposed pan-cancer prognostic signature (part 1 of 5).*

number	signature	cancer type	probeset	gene symbol
1	sigCangelosi2020	neuroblastoma	200738_s_at	PGK1
2	sigCangelosi2020	neuroblastoma	206686_at	PDK1
3	sigCangelosi2020	neuroblastoma	217356_s_at	PGK1
4	sigCangelosi2020	neuroblastoma	223172_s_at	MTFP1
5	sigCangelosi2020	neuroblastoma	223193_x_at	FAM162A
6	sigCangelosi2020	neuroblastoma	224314_s_at	EGLN1
7	sigCangelosi2020	neuroblastoma	226452_at	PDK1
8	sigCangelosi2020	neuroblastoma	230630_at	AK4
9	sigCangelosi2020	neuroblastoma	202022_at	ALDOC
10	sigChen2012	prostate cancer	207016_s_at	ALDH1A2
11	sigChen2012	prostate cancer	208490_x_at	AL031777.2
12	sigChen2012	prostate cancer	208490_x_at	HIST1H2BF
13	sigChen2012	prostate cancer	209487_at	RBPMS
14	sigChen2012	prostate cancer	213293_s_at	TRIM22
15	sigChen2012	prostate cancer	214527_s_at	PQBP1
16	sigChen2012	prostate cancer	221523_s_at	RRAGD
17	sigChen2012	prostate cancer	221667_s_at	HSPB8
18	sigGyorffy2013	lung cancer	201243_s_at	ATP1B1
19	sigGyorffy2013	lung cancer	202490_at	IKBKAP
20	sigGyorffy2013	lung cancer	202707_at	UMPS
21	sigGyorffy2013	lung cancer	202814_s_at	HEXIM1
22	sigGyorffy2013	lung cancer	203001_s_at	STMN2
23	sigGyorffy2013	lung cancer	203147_s_at	TRIM14
24	sigGyorffy2013	lung cancer	204179_at	MB
25	sigGyorffy2013	lung cancer	204584_at	L1CAM
26	sigGyorffy2013	lung cancer	205386_s_at	MDM2
27	sigGyorffy2013	lung cancer	206426_at	MLANA
28	sigGyorffy2013	lung cancer	208399_s_at	EDN3
29	sigGyorffy2013	lung cancer	210016_at	MYT1L
30	sigGyorffy2013	lung cancer	218881_s_at	FOSL2
31	sigGyorffy2013	lung cancer	219171_s_at	ZNF236
32	sigGyorffy2013	lung cancer	221591_s_at	FAM64A
33	sigHallett2012	breast cancer	201022_s_at	DSTN
34	sigHallett2012	breast cancer	201217_x_at	RPL3P7
35	sigHallett2012	breast cancer	201217_x_at	RPL3P4
36	sigHallett2012	breast cancer	201217_x_at	RPL3
37	sigHallett2012	breast cancer	203072_at	MYO1E
38	sigHallett2012	breast cancer	204338_s_at	RGS4
39	sigHallett2012	breast cancer	204544_at	HPS5
40	sigHallett2012	breast cancer	208089_s_at	TDRD3
41	sigHallett2012	breast cancer	208538_at	ANP32C
42	sigHallett2012	breast cancer	208885_at	LCP1
43	sigHallett2012	breast cancer	211073_x_at	RPL3P4
44	sigHallett2012	breast cancer	211073_x_at	RPL3
45	sigHallett2012	breast cancer	212039_x_at	RPL3P4
46	sigHallett2012	breast cancer	212039_x_at	RPL3
47	sigHallett2012	breast cancer	216143_at	PIAS1
48	sigHallett2012	breast cancer	217434_at	MC2R
49	sigHallett2012	breast cancer	220719_at	RP11-197N18.8
50	sigHallett2012	breast cancer	221306_at	GPR27

All the probeset ID's are based on the Affymetrix GPL96, GPL97, or GPL570 platforms. We retrieved the gene symbols through BioMart [25], BioGPS [34], or geneExpressionFromGEO [33]. The other parts can be found in Table S2, in Table S3, in Table S4, and in Table S5. The number of gene symbols for each signature can be different from the number of probesets because some probesets can correspond to multiple gene symbols and some other probesets might not correspond to any gene symbol.

Table S2: *Our proposed pan-cancer prognostic signature (part 2 of 5).*

number	signature	cancer type	probeset	gene symbol
51	sigVanLaar2010	colorectal cancer	1553954_at	ALG14
52	sigVanLaar2010	colorectal cancer	1554078_s_at	DNAJA3
53	sigVanLaar2010	colorectal cancer	1555832_s_at	KLF6
54	sigVanLaar2010	colorectal cancer	1555950_a_at	CD55
55	sigVanLaar2010	colorectal cancer	1560089_at	SLC25A25-AS1
56	sigVanLaar2010	colorectal cancer	1560587_s_at	PRDX5
57	sigVanLaar2010	colorectal cancer	1563796_s_at	EARS2
58	sigVanLaar2010	colorectal cancer	200006_at	PARK7
59	sigVanLaar2010	colorectal cancer	200632_s_at	NDRG1
60	sigVanLaar2010	colorectal cancer	200665_s_at	SPARC
61	sigVanLaar2010	colorectal cancer	200827_at	PLOD1
62	sigVanLaar2010	colorectal cancer	200838_at	CTSB
63	sigVanLaar2010	colorectal cancer	200839_s_at	CTSB
64	sigVanLaar2010	colorectal cancer	200931_s_at	VCL
65	sigVanLaar2010	colorectal cancer	200983_x_at	CD59
66	sigVanLaar2010	colorectal cancer	201012_at	ANXA1
67	sigVanLaar2010	colorectal cancer	201141_at	GPNMB
68	sigVanLaar2010	colorectal cancer	201170_s_at	BHLHE40
69	sigVanLaar2010	colorectal cancer	201185_at	HTRA1
70	sigVanLaar2010	colorectal cancer	201261_x_at	BGN
71	sigVanLaar2010	colorectal cancer	201289_at	CYR61
72	sigVanLaar2010	colorectal cancer	201323_at	EBNA1BP2
73	sigVanLaar2010	colorectal cancer	201422_at	IFI30
74	sigVanLaar2010	colorectal cancer	201422_at	PIK3R2
75	sigVanLaar2010	colorectal cancer	201426_s_at	VIM
76	sigVanLaar2010	colorectal cancer	201578_at	PODXL
77	sigVanLaar2010	colorectal cancer	201590_x_at	ANXA2
78	sigVanLaar2010	colorectal cancer	201666_at	TIMP1
79	sigVanLaar2010	colorectal cancer	201925_s_at	CD55
80	sigVanLaar2010	colorectal cancer	201926_s_at	CD55
81	sigVanLaar2010	colorectal cancer	201939_at	PLK2
82	sigVanLaar2010	colorectal cancer	201951_at	ALCAM
83	sigVanLaar2010	colorectal cancer	202068_s_at	LDLR
84	sigVanLaar2010	colorectal cancer	202237_at	AP002518.1
85	sigVanLaar2010	colorectal cancer	202237_at	NNMT
86	sigVanLaar2010	colorectal cancer	202238_s_at	NNMT
87	sigVanLaar2010	colorectal cancer	202419_at	KDSR
88	sigVanLaar2010	colorectal cancer	202457_s_at	PPP3CA
89	sigVanLaar2010	colorectal cancer	202478_at	TRIB2
90	sigVanLaar2010	colorectal cancer	202839_s_at	NDUFB7
91	sigVanLaar2010	colorectal cancer	202887_s_at	DDIT4
92	sigVanLaar2010	colorectal cancer	202904_s_at	LSM5
93	sigVanLaar2010	colorectal cancer	202939_at	ZMPSTE24
94	sigVanLaar2010	colorectal cancer	202949_s_at	FHL2
95	sigVanLaar2010	colorectal cancer	203083_at	THBS2
96	sigVanLaar2010	colorectal cancer	203382_s_at	APOE
97	sigVanLaar2010	colorectal cancer	203476_at	TPBG
98	sigVanLaar2010	colorectal cancer	203895_at	PLCB4
99	sigVanLaar2010	colorectal cancer	204264_at	CPT2
100	sigVanLaar2010	colorectal cancer	204472_at	GEM

All the probeset ID's are based on the Affymetrix GPL96, GPL97, or GPL570 platforms. The other parts of the signature can be found in Table S1, in Table S3, in Table S4, and in Table S5. The number of gene symbols for each signature can be different from the number of probesets because some probesets can correspond to multiple gene symbols and some other probesets might not correspond to any gene symbol.

Table S3: *Our proposed pan-cancer prognostic signature (part 3 of 5)*

number	signature	cancer type	probeset	gene symbol
101	sigVanLaar2010	colorectal cancer	204620_s_at	VCAN
102	sigVanLaar2010	colorectal cancer	204679_at	KCNK1
103	sigVanLaar2010	colorectal cancer	205677_s_at	DLEU1_2
104	sigVanLaar2010	colorectal cancer	205677_s_at	DLEU1
105	sigVanLaar2010	colorectal cancer	205963_s_at	DNAJA3
106	sigVanLaar2010	colorectal cancer	207543_s_at	P4HA1
107	sigVanLaar2010	colorectal cancer	207574_s_at	GADD45B
108	sigVanLaar2010	colorectal cancer	208891_at	DUSP6
109	sigVanLaar2010	colorectal cancer	208892_s_at	DUSP6
110	sigVanLaar2010	colorectal cancer	208893_s_at	DUSP6
111	sigVanLaar2010	colorectal cancer	208918_s_at	NADK
112	sigVanLaar2010	colorectal cancer	208961_s_at	KLF6
113	sigVanLaar2010	colorectal cancer	209043_at	PAPSS1
114	sigVanLaar2010	colorectal cancer	209101_at	CTGF
115	sigVanLaar2010	colorectal cancer	209184_s_at	IRS2
116	sigVanLaar2010	colorectal cancer	209185_s_at	IRS2
117	sigVanLaar2010	colorectal cancer	209193_at	PIM1
118	sigVanLaar2010	colorectal cancer	209345_s_at	PI4K2A
119	sigVanLaar2010	colorectal cancer	209386_at	TM4SF1
120	sigVanLaar2010	colorectal cancer	209387_s_at	TM4SF1
121	sigVanLaar2010	colorectal cancer	209457_at	DUSP5
122	sigVanLaar2010	colorectal cancer	209545_s_at	RIPK2
123	sigVanLaar2010	colorectal cancer	209624_s_at	MCCC2
124	sigVanLaar2010	colorectal cancer	209711_at	SLC35D1
125	sigVanLaar2010	colorectal cancer	209875_s_at	SPP1
126	sigVanLaar2010	colorectal cancer	210095_s_at	IGFBP3
127	sigVanLaar2010	colorectal cancer	210275_s_at	ZFAND5
128	sigVanLaar2010	colorectal cancer	210427_x_at	ANXA2
129	sigVanLaar2010	colorectal cancer	210495_x_at	FN1
130	sigVanLaar2010	colorectal cancer	210512_s_at	VEGFA
131	sigVanLaar2010	colorectal cancer	210517_s_at	AKAP12
132	sigVanLaar2010	colorectal cancer	210592_s_at	SAT1
133	sigVanLaar2010	colorectal cancer	210652_s_at	TTC39A
134	sigVanLaar2010	colorectal cancer	210845_s_at	PLAUR
135	sigVanLaar2010	colorectal cancer	211074_at	CH507-513H4.3
136	sigVanLaar2010	colorectal cancer	211074_at	CH507-513H4.5
137	sigVanLaar2010	colorectal cancer	211074_at	CH507-513H4.6
138	sigVanLaar2010	colorectal cancer	211074_at	CH507-513H4.4
139	sigVanLaar2010	colorectal cancer	211719_x_at	FN1
140	sigVanLaar2010	colorectal cancer	211924_s_at	PLAUR
141	sigVanLaar2010	colorectal cancer	211928_at	DYNC1H1
142	sigVanLaar2010	colorectal cancer	211988_at	RP5-1028K7.3
143	sigVanLaar2010	colorectal cancer	211988_at	SMARCE1
144	sigVanLaar2010	colorectal cancer	212013_at	PXDN
145	sigVanLaar2010	colorectal cancer	212143_s_at	IGFBP3
146	sigVanLaar2010	colorectal cancer	212171_x_at	VEGFA
147	sigVanLaar2010	colorectal cancer	212463_at	CD59
148	sigVanLaar2010	colorectal cancer	212464_s_at	FN1
149	sigVanLaar2010	colorectal cancer	212501_at	CEBPB
150	sigVanLaar2010	colorectal cancer	212632_at	STX7

All the probeset ID's are based on the Affymetrix GPL96, GPL97, or GPL570 platforms. The other parts of the signature can be found in Table S1, in Table S2, in Table S4, and in Table S5. The number of gene symbols for each signature can be different from the number of probesets because some probesets can correspond to multiple gene symbols and some other probesets might not correspond to any gene symbol.

Table S4: *Our proposed pan-cancer prognostic signature (part 4 of 5).*

number	signature	cancer type	probeset	gene symbol
151	sigVanLaar2010	colorectal cancer	212884_x_at	APOE
152	sigVanLaar2010	colorectal cancer	213274_s_at	CTSB
153	sigVanLaar2010	colorectal cancer	213503_x_at	ANXA2
154	sigVanLaar2010	colorectal cancer	213905_x_at	BGN
155	sigVanLaar2010	colorectal cancer	214581_x_at	TNFRSF21
156	sigVanLaar2010	colorectal cancer	214620_x_at	PAM
157	sigVanLaar2010	colorectal cancer	214866_at	PLAUR
158	sigVanLaar2010	colorectal cancer	215033_at	TM4SF1
159	sigVanLaar2010	colorectal cancer	215034_s_at	TM4SF1
160	sigVanLaar2010	colorectal cancer	215792_s_at	DNAJC11
161	sigVanLaar2010	colorectal cancer	216392_s_at	SEC23IP
162	sigVanLaar2010	colorectal cancer	216442_x_at	FN1
163	sigVanLaar2010	colorectal cancer	217762_s_at	RAB31
164	sigVanLaar2010	colorectal cancer	217773_s_at	NDUFA4
165	sigVanLaar2010	colorectal cancer	217996_at	PHLDA1
166	sigVanLaar2010	colorectal cancer	217996_at	NAP1L1
167	sigVanLaar2010	colorectal cancer	218213_s_at	TMEM258
168	sigVanLaar2010	colorectal cancer	218698_at	RP11-196I18.3
169	sigVanLaar2010	colorectal cancer	218698_at	APIP
170	sigVanLaar2010	colorectal cancer	218856_at	TNFRSF21
171	sigVanLaar2010	colorectal cancer	218902_at	NOTCH1
172	sigVanLaar2010	colorectal cancer	219038_at	MORC4
173	sigVanLaar2010	colorectal cancer	219206_x_at	TMBIM4
174	sigVanLaar2010	colorectal cancer	219206_x_at	TMBIM4
175	sigVanLaar2010	colorectal cancer	219539_at	GEMIN6
176	sigVanLaar2010	colorectal cancer	221419_s_at	CH507-513H4.3
177	sigVanLaar2010	colorectal cancer	221419_s_at	CH507-513H4.6
178	sigVanLaar2010	colorectal cancer	221419_s_at	CH507-513H4.4
179	sigVanLaar2010	colorectal cancer	221479_s_at	BNIP3L
180	sigVanLaar2010	colorectal cancer	221563_at	DUSP10
181	sigVanLaar2010	colorectal cancer	221648_s_at	AGMAT
182	sigVanLaar2010	colorectal cancer	221656_s_at	ARHGEF10L
183	sigVanLaar2010	colorectal cancer	221730_at	COL5A2
184	sigVanLaar2010	colorectal cancer	221731_x_at	VCAN
185	sigVanLaar2010	colorectal cancer	221745_at	DCAF7
186	sigVanLaar2010	colorectal cancer	222421_at	UBE2H
187	sigVanLaar2010	colorectal cancer	222994_at	PRDX5
188	sigVanLaar2010	colorectal cancer	223003_at	TRIR
189	sigVanLaar2010	colorectal cancer	223122_s_at	SFRP2
190	sigVanLaar2010	colorectal cancer	223163_s_at	ZC3HC1
191	sigVanLaar2010	colorectal cancer	223312_at	PRADC1
192	sigVanLaar2010	colorectal cancer	223454_at	CXCL16
193	sigVanLaar2010	colorectal cancer	223455_at	TCHP
194	sigVanLaar2010	colorectal cancer	224602_at	C4orf3
195	sigVanLaar2010	colorectal cancer	224606_at	KLF6
196	sigVanLaar2010	colorectal cancer	224657_at	ERRFI1
197	sigVanLaar2010	colorectal cancer	224777_s_at	PAFAH1B2
198	sigVanLaar2010	colorectal cancer	224806_at	TRIM25
199	sigVanLaar2010	colorectal cancer	224890_s_at	LAMTOR4
200	sigVanLaar2010	colorectal cancer	224911_s_at	DCBLD2

All the probeset ID's are based on the Affymetrix GPL96, GPL97, or GPL570 platforms. The other parts of the signature can be found in Table S1, in Table S2, in Table S3, and in Table S5. The number of gene symbols for each signature can be different from the number of probesets because some probesets can correspond to multiple gene symbols and some other probesets might not correspond to any gene symbol.

Table S5: ***Our proposed pan-cancer prognostic signature (part 5 of 5).***

number	signature	cancer type	probeset	gene symbol
201	sigVanLaar2010	colorectal cancer	225010_at	CCDC6
202	sigVanLaar2010	colorectal cancer	225011_at	PRKAR2A
203	sigVanLaar2010	colorectal cancer	225337_at	ABHD2
204	sigVanLaar2010	colorectal cancer	225494_at	DYNLL2
205	sigVanLaar2010	colorectal cancer	225670_at	ATPSCKMT
206	sigVanLaar2010	colorectal cancer	225750_at	ERO1A
207	sigVanLaar2010	colorectal cancer	226041_at	NAPEPLD
208	sigVanLaar2010	colorectal cancer	226594_at	ENTPD5
209	sigVanLaar2010	colorectal cancer	226648_at	HIF1AN
210	sigVanLaar2010	colorectal cancer	226727_at	CISD3
211	sigVanLaar2010	colorectal cancer	226987_at	RBM15B
212	sigVanLaar2010	colorectal cancer	227143_s_at	BID
213	sigVanLaar2010	colorectal cancer	227338_at	LOC440983
214	sigVanLaar2010	colorectal cancer	227735_s_at	C10orf99
215	sigVanLaar2010	colorectal cancer	227736_at	C10orf99
216	sigVanLaar2010	colorectal cancer	227961_at	CTSB
217	sigVanLaar2010	colorectal cancer	229676_at	MTPAP
218	sigVanLaar2010	colorectal cancer	231576_at	Q9H741
219	sigVanLaar2010	colorectal cancer	234983_at	SPRING1
220	sigVanLaar2010	colorectal cancer	241355_at	HR
221	sigVanLaar2010	colorectal cancer	242648_at	KLHL8
222	sigVanLaar2010	colorectal cancer	35156_at	R3HCC1
223	sigVanLaar2010	colorectal cancer	36711_at	MAFF
224	sigVanLaar2010	colorectal cancer	58780_s_at	ARHGEF40

All the probeset ID's are based on the Affymetrix GPL96, GPL97, or GPL570 platforms. The other parts of the signature can be found in Table S1, in Table S2, in Table S3, and in Table S4. The number of gene symbols for each signature can be different from the number of probesets because some probesets can correspond to multiple gene symbols and some other probesets might not correspond to any gene symbol.

Table S6: ***The sigNagy2021 pan-cancer prognostic signature.***

number	probeset	gene symbol
1	202628_s_at	SERPINE1
2	204947_at	E2F1
3	209696_at	FBP1
4	205767_at	EREG
5	203213_at	CDK1
6	204531_s_at	BRCA1
7	202431_s_at	MYC
8	209360_s_at	RUNX1

The authors provided the original signature as a list of gene symbols [16], so we used their tool Jetset [3] to retrieve the most appropriate probesets for the HG-U133A (GPL96) platform.

Table S7: ***The sigYu2021 pan-cancer prognostic signature.***

number	probeset	gene symbol
1	202628_s_at	SERPINE1
2	204943_at	ADAM12
3	204475_at	MMP1
4	202185_at	PLOD3
5	228703_at	P4HA3

The authors provided the original signature as a list of gene symbols [13], so we used Jetset [3] to retrieve the most appropriate probesets for the HG-U133A (GPL96) platform. Jetset did not associate any probeset to the P4HA3 gene, so we employed BioGPS [34] to this end.

Figure S1: ***Components of our pan-cancer prognostic signature.***

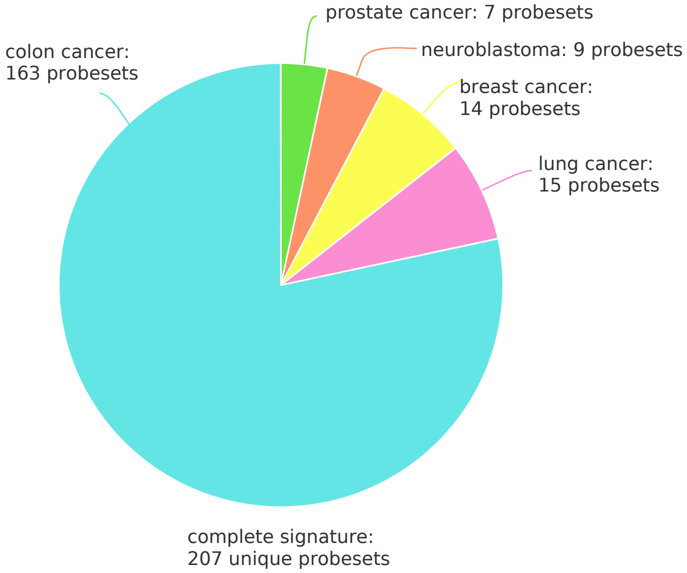

Pie-chart representing the contribution of the singular signatures to our pan-cancer signature. Neuroblastoma: sigCangelosi2020 signature [27], that is 4.33% of the our pan-cancer signature. Prostate cancer: sigChen2012 signature [28], that is 3.37% of the our pan-cancer signature. Lung cancer: sigGyorffy2013 signature [29], that is 7.21% of the our pan-cancer signature. Breast cancer: sigHallett2012 signature [30], that is 6.73% of the our pan-cancer signature. Colon cancer: sigVanLaar2010 signature [31, 32], that is 78.37% of the our pan-cancer signature. The complete signature contains 208 probesets, including 207 probesets present only once and the 203072_at probeset (MYO1E gene) present twice.

Figure S2: ***Key-term enrichment analysis of protein products of genes in the combination signature.***

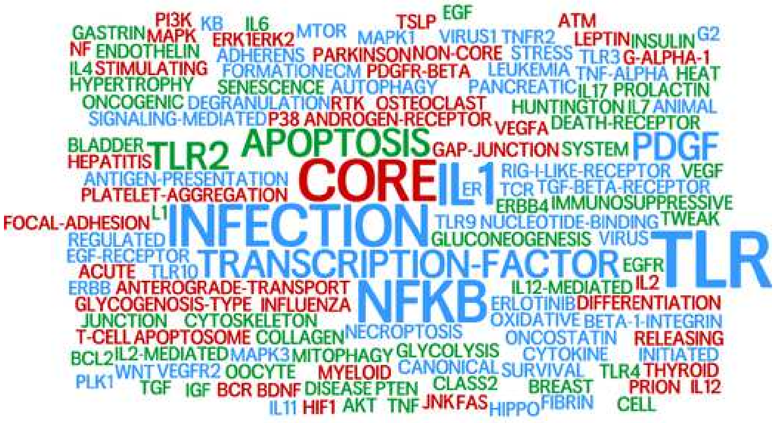

Size of different key-terms is proportional with -log of statistical significance of appearance of each key-term in title of enriched pathways. We generated this image with pathDIP [57].

Binary statistical rates

List of statistical rates to evaluate confusion matrices and their formulas:

$$\text{MCC} = \frac{TP \cdot TN - FP \cdot FN}{\sqrt{(TP+FP)\cdot(TP+FN)\cdot(TN+FP)\cdot(TN+FN)}} \tag{1}$$

(worst value $= -1$; best value $= +1$)

$$\text{normMCC} = \frac{MCC+1}{2} \tag{2}$$

(worst value $= 0$; best value $= 1$)

$$\text{F}_1 \text{ score} = \frac{2 \cdot TP}{2 \cdot TP + FP + FN} \tag{3}$$

(worst value $= 0$; best value $= 1$)

$$\text{accuracy} = \frac{TP + TN}{TP + FN + TN + FP} \tag{4}$$

(worst value $= 0$; best value $= 1$)

$$\text{true positive rate, recall, sensitivity} = \frac{TP}{TP + FN} \tag{5}$$

(worst value $= 0$; best value $= 1$)

$$\text{true negative rate, specificity} = \frac{TN}{TN + FP} \tag{6}$$

(worst value $= 0$; best value $= 1$)

$$\text{positive predictive value, precision} = \frac{TP}{TP + FP} \tag{7}$$

(worst value $= 0$; best value $= 1$)

$$\text{negative predictive value} = \frac{TN}{TN + FN} \tag{8}$$

(worst value $= 0$; best value $= 1$)

$$\text{Precision-Recall (PR) curve} = \begin{cases} \textit{true positive rate} & \textit{on the x axis} \\ \textit{precision} & \textit{on the y axis} \end{cases} \tag{9}$$

$$(\text{worst value} = 0; \text{ best value} = 1)$$

$$\text{ROC curve} = \begin{cases} false\ positive\ rate & on\ the\ x\ axis \\ true\ positive\ rate & on\ the\ y\ axis \end{cases} \tag{10}$$

$$(\text{worst value} = 0; \text{ best value} = 1)$$
